# Reactivation of occult HBV infection in an HIV/HCV Co-infected patient successfully treated with sofosbuvir/ledipasvir: a case report and review of the literature

**DOI:** 10.1186/s12879-017-2287-y

**Published:** 2017-03-01

**Authors:** Gabriele Fabbri, Ilaria Mastrorosa, Alessandra Vergori, Valentina Mazzotta, Carmela Pinnetti, Susanna Grisetti, Mauro Zaccarelli, Adriana Ammassari, Andrea Antinori

**Affiliations:** National Institute of Infectious Diseases “Lazzaro Spallanzani”, Via Portuense 292, 00152 Rome, Italy

**Keywords:** Acute hepatitis, Sofosbuvir/ledipasvir, HBV reactivation, HIV, DAA

## Abstract

**Background:**

Reactivation of occult or inactive Hepatitis B virus (HBV) infection during immunosuppressant treatments is well known and widely described in literature. The same observation has been made in Hepatitis C (HCV)-infected patients previously exposed to HBV and treated with interferon-free DAA treatments. Because of common transmission routes, persons may have been exposed to HCV, HBV and HIV, but few cases have been reported in this scenario to date. Frequency of HBV reactivation in HIV/HCV co-infected patients previously exposed to HBV and treated with DAA remains unclear. Herein, we report an episode of HBV reactivation in an HIV/HCV co-infected patient prescribed with sofosbuvir/ledipasvir for HCV.

**Case presentation:**

The patient is a Caucasian 54-years old female, with HIV/HCV co-infection (genotype 4), and a previous exposure to HBV, documented by negativity of HBsAg and positivity of HBsAb and HBcAb. Her medical history included: myocardial infarct, chronic kidney disease stage 3, chronic obstructive pulmonary disease, and mild pulmonary hypertension. HCV had not been treated with interferon (IFN)-based regimens and liver stiffness was 10.5 KPa (Metavir stage F3) at hepatic elastography. Because of CKD, she was prescribed with a nucleoside reverse transcriptase (NRTI)-sparing regimen including darunavir/ritonavir plus etravirine, and thereafter with sofosbuvir/ledipasvir for 12 weeks. Four weeks after DAA termination, the patient was hospitalized with symptoms of acute hepatitis. Blood tests showed HCV RNA <12 IU/ml, but positivity of HBAg, HBeAg, and of anti-core antibodies (IgM and IgG), while anti-HBs and anti-HBe antibodies were negative. HBV DNA was 6.06 Log_10_ IU/ml. Entecavir was started obtaining resolution of symptoms, normalization of liver enzymes, as well as reduction of HBV DNA and of quantitative HBV surface antigen.

**Conclusions:**

This case-report highlights the risk of HBV reactivation with interferon-free DAA treatment in HIV/HCV co-infected patients previously exposed to HBV and who have contraindications for treatment with nucleoside/nucleotide reverse transcriptase Inhibitors because of comorbid conditions. In the setting of HIV infection, clinicians prescribing DAA should be aware of this risk, and HBV assessment at treatment start as well as virological monitoring during DAA treatment is recommended. Large epidemiological and virological studies are needed to investigate reactivation of occult HBV infection more in depth.

## Background

Reactivation of HBV infection during immunosuppressant treatments is well known and widely described in literature [1]. The risk concerns subjects with occult HBV infection (HBV DNA detected in serum or in the liver in HBsAg-negative patients with or without serologic markers of previous viral exposure) or inactive HBV chronic carriers (HBsAg-positive with normal ALT and HBV DNA <3.30 log_10_ IU/ml), either undergoing transplant or treated with chemotherapy or immunosuppressant drugs for hematologic malignancies or rheumatologic disorders [2].

In the past, with the use of interferon (IFN)-based HCV therapy, exacerbation of acute HBV hepatitis in HBV-exposed patients has been described and an immune modulating role of IFN postulated [3]. More recently, a similar observation has been made with the use of interferon-free directly acting antivirals (DAA) for HCV treatment [4]. In HIV/HCV co-infected patients, who show HCV cure rates comparable to the general population [5], only very few cases of HBV reactivation have been reported [6–10]. Thus, the frequency of HBV reactivation in HIV/HCV co-infected patients previously exposed to HBV and treated with DAA remains unclear.

Herein, we report an episode of reactivation of occult HBV infection in an HIV/HCV co-infected patient prescribed with sofosbuvir/ledipasvir for HCV treatment. Further, a short review of similar cases published in literature is outlined.

## Case presentation

**Fig. 1:**
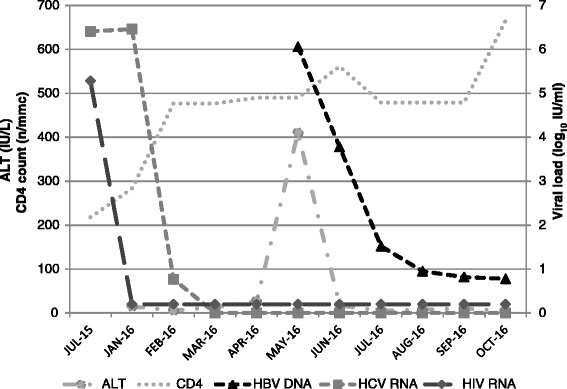
Plasma HIV, HCV, HBV viral loads, liver function and CD4 cell count depicted over time

The patient is a Caucasian 54-years old female diagnosed with HIV in 1986 and with chronic HCV hepatitis (genotype 4) in 1992. For many years, she was lost to follow-up and refused antiretroviral therapy presenting only once in 2011, when blood tests showed HIV RNA 4.93 log_10_ IU/ml, CD4 cells 245/mmc. Concurrently, previous exposure to HBV infection was documented: 12.2 mIU/ml HBsAb (positive >10 mIU/ml), positive HBcAb, and negative HBsAg at 0.01 IU/ml (positive >0.05 IU/ml). Afterwards she showed up again in June 2015, when viroimmunological exams showed: HIV RNA 5.28 log_10_ copies/ml and CD4 count 218/mmc. At that time, her medical history included: myocardial infarct, chronic kidney disease (CKD) stage 3, chronic obstructive pulmonary disease, and mild pulmonary hypertension. HCV had not been treated with IFN-based regimens and liver stiffness was 10.5 KPa (Metavir stage F3) at hepatic elastography. Because of CKD, first-line antiretroviral treatment was a nucleoside reverse transcriptase (NRTI)-sparing regimen including darunavir/ritonavir 800/100 mg plus etravirine 400 mg QD. In January 2016, blood tests showed HIV RNA not detected <40 copies/ml with CD4 cells 283/mmc and treatment with sofosbuvir/ledipasvir 400/90 mg once daily was prescribed for 12 weeks. In May 2016, four weeks after treatment completion, the patient presented with jaundice reporting vomiting, nausea, and abdominal pain. She was admitted to hospital, and an elevation of liver enzymes (ALT 435 IU/l and AST 410 IU/l, respectively) and total bilirubin at 7.1 mg/dl were documented. HCV RNA was <12 IU/ml, HIV RNA <40 copies/ml and CD4 count had increased to 561/mmc. With regards to HBV markers, HBsAg (3.71 log_10_ IU/ml) and HBeAg changed into positive together with IgM and IgG HBcAb, while HBsAb and HBeAb remained negative. Furthermore, HBV DNA was 6.06 log_10_ IU/ml. Treatment with entecavir 0.5 mg once daily was promptly started obtaining resolution of symptoms. One month later, blood tests documented normalization of transaminases and reduction of HBV DNA at 3.78 log_10_ IU/l, of HBsAg 1.91 log_10_ IU/ml and negativization of HBeAg (Fig. 1). Treatment for HBV infection is still ongoing without adverse events and sustained virological response for HCV was achieved at 12 and at 24 weeks of observation.

## Discussion

HBV reactivation of occult or inactive HBV infection in HCV-infected persons during or after DAA therapy is considered a rare event, even though lately an important warning by the FDA about 24 cases of HBV reactivation during DAA HCV treatment has been released [11]. In literature to date, only six cases of these have been reported and characteristics are summarized in Table 1: all events occurred in subjects aged 50 years or more, mostly infected by HCV genotype 1, with frequent previous IFN exposure, and treated with sofobuvir- or daclatasvir-based DAA regimens. Notably, only one case referred to a person with HIV co-infection. Occasionally HBV reactivation occurred during DAA treatment, while otherwise it developed after treatment completion. In some cases, diagnosis was retrospectively established.

**Table 1:** Characteristics of patients with HBV reactivation during or after DAA treatment for HCV published in literature

Patient	Ref.	Gender, age	HCV genotype	Previous IFN	DAA treatment	HBV profile	HIV status	Symptoms onset
1	[7]	M, 53	4d	Yes	SOF/LDV	Resolved HBV	+	At 6 weeks during DAA
2	[6]	M, 55	1a	Yes	SOF/SMV	Inactive HBV	-	At 8 weeks during DAA
3	[6]	M, 57	1a	Yes	SOF/SMV	Occult HBV	-	At 4 weeks during DAA
4	[10]	F, 59	1b	Yes	SOF/SMV/RBV	Resolved HBV	-	At 11 weeks during DAA
5	[9]	M, 69	1b	No	DCV/ASV	Inactive HBV	-	At 6 weeks during DAA
6	[8]	F, 83	1b	NA	DCV/ASV	NA (HBsAg neg)	-	At 20 weeks after DAA

Legend: *M* male, *F* female, *IFN* interferon, *RBV* ribavirin, *SOF* sofosbuvir, *SMV* simeprevir, *DCV* daclatasvir, *ASV* asunaprevir, *HBV* hepatitis B virus, *HCV* hepatitis C virus, *DAA* direct antiviral agents, *NA* not available, *neg* negative

To date, risk of HBV reactivation during treatment with ledipasvir/sofosbuvir seems low, and our patient is only the second case described in literature [7]. Regarding frequency of the event, reassuring data are available from a recent study by Sulkowski et al., which retrospectively reanalyzed HBV markers in serum samples of 173 HCV-infected patients without active HBV or HIV infection and treated with a combination of ledipasvir/sofosbuvir. Notably, HBV reactivation during or after HCV clearance was found in none out of the 103 previously HBV-exposed patients [12]. Differently, in patients with HCV and HBV co-infection, transitory HBV DNA reactivation rate seems very high, reaching 88% of a small case series treated with ledipasvir/sofosbuvir [13]. Since accurate information regarding risk of HBV reactivation in patients undergoing DAA therapy is lacking, an important prospective study is ongoing in patients with active HBV/HCV infection [13], but the issue should also be addressed in HCV-infected patients with occult HBV infection.

In our patient, the rapid clearance of HCV RNA with DAA treatment could have triggered HBV reactivation leading to acute symptomatic hepatitis B. It also should to be noted that, the low levels of HBsAb in 2011 and the absence of this protective marker at hepatitis onset, might have played an important role in allowing HBV reactivation. In fact, our patient was not taking any ARV regimen for 15 years after HIV diagnosis and this has led to marked immunodeficiency: similarly to what happens in patients undergoing allogenic stem cells transplantation, we can assume that she may have lost her immunity against HBV [14].

The molecular mechanisms involved in HCV/HBV interferences are controversial and incompletely understood. It seems that HBV can be chronically suppressed by HCV infection with alternate phases of dominance of one virus on the other [15, 16] and a suppressing effect of HCV core proteins on HBV replication has been postulated in some studies [17, 18]. Other studies have suggested that, host genes and immune regulation, such as kinase pathways or microRNA pathways, mediate the mechanism of underlying HBV inhibition [19, 20]. Regardless of the molecular mechanisms involved in HCV/HBV co-infection, the introduction of DAA drugs that are specifically directed against HCV without inhibitory effect on HBV may unbalance viral and/or host interactions and eventually allow HBV reactivation [21].

Our case report poses some further questions, because the patient had HBV reactivation after DAA treatment, but also was HIV-positive making the scenario even more complex. On one side, HIV-infected patients may experience various levels of immune deficiency, because of lower CD4 cell count and immune dysregulation [22], malignancies or rheumatologic diseases. Also immune reconstitution in antiretroviral-treated patients may play a role in the same direction. In fact, reactivation of several latent infections, including HBV infection, is facilitated by immune reconstitution [23], and our patient experienced a relevant increase in CD4 cell count when comparing values before and after DAA treatment. On the contrary however, it is likely that a considerable proportion of patients with HIV infection will receive anti-HBV agents, like lamivudine, emtricitabine or tenofovir, as part of their antiretroviral therapy during DAA therapy, and therefore will be protected from HBV reactivation. Nonetheless, this may not apply to a considerable number of HIV-positive patients, that have contraindications for treatment with nucleoside/nucleotide reverse transcriptase Inhibitors because of comorbid conditions and who receive dual regimens [24].

## Conclusions

In conclusion, this case-report highlights the risk of HBV reactivation with interferon-free DAA treatment in HIV/HCV co-infected patients previously exposed to HBV and who have contraindications for treatment with nucleoside/nucleotide reverse transcriptase Inhibitors because of comorbid conditions. In the setting of HIV infection, clinicians prescribing DAA should be aware of this risk, and HBV assessment at treatment start as well as virological monitoring during DAA treatment is recommended. Large epidemiological and virological studies are needed to investigate reactivation of occult HBV infection more in depth.

## Abbreviations

CKD: chronic kidney disease
DAA: direct antiviral agents
DAA: direct antiviral agents
IFN: interferon
NRTI: nucleoside reverse transcriptase inhibitors
